# New intelligent multifunctional SiO_2_/VO_2_ composite films with enhanced infrared light regulation performance, solar modulation capability, and superhydrophobicity

**DOI:** 10.1080/14686996.2017.1360752

**Published:** 2017-08-22

**Authors:** Chao Wang, Li Zhao, Zihui Liang, Binghai Dong, Li Wan, Shimin Wang

**Affiliations:** ^a^ Hubei Collaborative Innovation Center for Advanced Organic Chemical Materials, Wuhan, PR China; ^b^ Faculty of Materials Science and Engineering, Ministry of Education Key Laboratory for the Green Preparation and Application of Functional Materials, Hubei University, Wuhan, PR China

**Keywords:** Intelligent SiO_2_/VO_2_ composite films, IR-regulating, anti-oxidation, anti-acid, superhydrophobic, 10 Engineering and Structural materials, 103 Composites, 204 Optics / Optical applications, 306 Thin film / Coatings

## Abstract

Highly transparent, energy-saving, and superhydrophobic nanostructured SiO_2_/VO_2_ composite films have been fabricated using a sol–gel method. These composite films are composed of an underlying infrared (IR)-regulating VO_2_ layer and a top protective layer that consists of SiO_2_ nanoparticles. Experimental results showed that the composite structure could enhance the IR light regulation performance, solar modulation capability, and hydrophobicity of the pristine VO_2_ layer. The transmittance of the composite films in visible region (*T*_lum_) was higher than 60%, which was sufficient to meet the requirements of glass lighting. Compared with pristine VO_2_ films and tungsten-doped VO_2_ film, the near IR control capability of the composite films was enhanced by 13.9% and 22.1%, respectively, whereas their solar modulation capability was enhanced by 10.9% and 22.9%, respectively. The water contact angles of the SiO_2_/VO_2_ composite films were over 150°, indicating superhydrophobicity. The transparent superhydrophobic surface exhibited a high stability toward illumination as all the films retained their initial superhydrophobicity even after exposure to 365 nm light with an intensity of 160 mW^**.**^cm^−2^ for 10 h. In addition, the films possessed anti-oxidation and anti-acid properties. These characteristics are highly advantageous for intelligent windows or solar cell applications, given that they can provide surfaces with anti-fogging, rainproofing, and self-cleaning effects. Our technique offers a simple and low-cost solution to the development of stable and visible light transparent superhydrophobic surfaces for industrial applications.

## Graphical abstract

**Figure:**
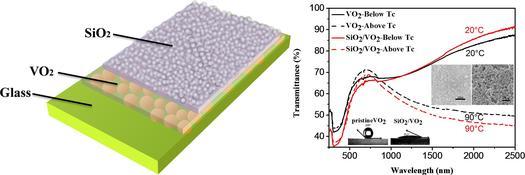

## 1. Introduction

The potential application of VO_2_ thin films to intelligent windows has received considerable attention because of the over-increasing consumption of primary energy sources in recent decades [1−3]. VO_2_ is a typical material with good thermal phase transition characteristics and has been widely used in intelligent windows [4]. VO_2_ can adjust the inflow of solar heat by switching transmittance in the infrared (IR) region (780–2500 nm) while maintaining visible transmittance. The crystal structure of VO_2_ is monoclinic (M phase) when its transition temperature (*T*_C_) is higher than room temperature. As IR light and visible light have higher transmittance, room temperature will gradually increase as light enters. However, the crystal structure of VO_2_ becomes tetragonal rutile (R phase) when its *T*_C_ is lower than room temperature. The transmittance of visible light remains constant, whereas the transmittance of IR light is reduced. Consequently, the heat generated by IR light cannot enter the room [5,6]. The aforementioned phenomenon allows selectively controlling different wavelengths of light entering a room according to indoor temperature. This process does not require any other forms of external energy to drive intelligent temperature control. VO_2_ is the first choice as material for intelligent temperature control due to the abrupt change in its optical properties before and after phase change. However, the main obstacles to the large-scale application of VO_2_ films include decreasing phase transition temperature [7,8], enhancing visible light transmittance [9], increasing solar conditioning capacity [10] under strong visible light absorption conditions [11], and maintaining long-term stability upon exposure to air for a long period [12,13] or under high temperatures (above 300 °C) [14].

Metal ion doping of VO_2_ thin films and the preparation of effective composite films are two main strategies for reducing phase transition temperature and improving transmittance, regulation rate, and anti-oxidant capacity of VO_2_ thin film. The phase transition temperature of VO_2_ thin films is effectively reduced to room temperature through ion doping. Metal ion doping of VO_2_ thin films is an effective approach to reduce *T*_C_. It mainly involves doping with cations and anions, such as tungsten [15,16], molybdenum [17], silicon [18], fluorine [19,20], niobium [21], and lanthanum [22]. Among these dopants, tungsten is most effective, lowering *T*_C_ by 20 °C per 1 at% [15]. VO_2_ thin films can be co-doped with mixed metal ions, such as tungsten and molybdenum [23], tungsten and magnesium [24], and tungsten and fluorine [25]. A VO_2_ thin film doped with lanthanum was prepared by Wang et al. Below 3 at% La doping level, the band gap (E_g_) decreased steadily with the increase of dopant concentration. The 4 at% La-doped sample showed the best combination of *T*_lum_ 50.1% and ∆*T*_sol_ 10.3%, compared with other reported VO_2_ continuous thin films [22]. Tungsten-doped VO_2_ films have been prepared by a sol–gel method in our group. The phase transition temperature was further reduced to 32 °C, which was sufficient to meet the practical requirements [26]. A composite film typically contains three layers: the buffer layer, the functional layer, and the reinforcement layer. The buffer layer is primarily designed to improve the mechanical properties of a film, including its adhesion to glass as well as its hardness and stress [27]. Si_3_N_4_ and TiO_2_ are the primary buffer layers used [28]. The functional layer refers to pristine or doped VO_2_ films that exhibit typical thermal phase transition properties. The reinforcement layer, which can also be called anti-reflective layer, is mainly designed to increase the transmittance of light [29] and improve anti-oxidation and anti-acid properties [30]. TiO_2_ [31], SiO_2_ [12], and ZrO_2_ [32] are the primary anti-reflective layers used. The low visible light transmittance of VO_2_ thin films is mainly due to their intrinsic absorption and light reflection. As the refractive index of VO_2_ thin films in the visible region and their reflectivity are high, the anti-reflective design of composite films must be enhanced.

Much research has been devoted to the fabrication of VO_2_-based composite films on glass or flexible substrates aiming to increase the visible range transmittance. However, progress toward the optimization of one factor always comes at the expense of the other. Yu et al. fabricated the SiO_2_/VO_2_ composite films with a *T*_lum_ improved from 37.6% to 47.7%, while ∆*T*_sol_ exhibited a decrease from 8.06% to 7.62% via radio frequency (RF) sputtering and chemical vapor deposition, and the near-infrared (NIR) control capability was obviously decreased as well [33]. VO_2_ thin film based on silicon–aluminum was prepared by Liu et al., in which the transmittance of the visible region was significantly enhanced (22% for *T*_lum_), whereas the NIR control capability was slightly decreased [34]. There were only a few reports on the fabrication of VO_2_-based composite films with enhanced solar conditioning capacity. Powell et al. fabricated the VO_2_/SiO_2_/TiO_2_ composite films by chemical vapor deposition. The deposition of the SiO_2_/TiO_2_ over-layer resulted in a dramatic improvement of visible light transmission whilst also doubling the solar modulation of the material. However, the highest visible light transmittance was still below 60%, which was not sufficient to meet the requirements of glass lighting [35]. In addition, nanoporous films [36], biomimetic moth-eye structures [37], and micro-grid structures [38] are three major nanostructuring approaches to enhance both *T*_lum_ and ∆*T*_sol_ of VO_2_ films. Micro-patterned VO_2_ films were fabricated by Lu et al. using a facile screen printing method; the best performing sample gave 43.3% *T*_lum_ and 14.9% ∆*T*_sol_, which were comparable to most approaches used to enhance thermochromic properties [38].

Superhydrophobic surfaces, such as composite films on glasses and metals, have many unique properties, such as self-cleaning, anti-sticking, anti-icing, anti-biofouling, water and bacteria proofing, drag reduction, and humidity proofing [39−43]. In the present study, intelligent multifunctional SiO_2_/VO_2_ composite films were fabricated using the sol–gel method. These composite films are composed of an underlying infrared (IR)-regulating VO_2_ layer and a top SiO_2_ protective layer that is deposited with SiO_2_ nanoparticles. They differ from a conventional approach, which includes a solid reinforcing layer. The accumulation of SiO_2_ nanoparticles in the film constitutes a highly porous nanoscale network structure, and the deposited SiO_2_ thin films has low surface energy, which renders excellent superhydrophobicity and self-cleaning properties. In addition, nanosilica with small particles provided a coarse micro-nanostructure by stacking the surfaces, thereby promoting reduction in the reflectivity of composite films. While maintaining the outstanding optical transmittance which was higher than 60% of VO_2_ film, the solar control ability of composite film is greatly improved. The experimental results showed an interesting phenomenon, i.e. both NIR control capability and solar modulation capability were simultaneously enhanced. This phenomenon provides a new avenue toward enhancing the visible light transmittance and solar energy regulation capacity of VO_2_ thin films. Our technique offers a simple and low-cost solution to the development of stable and highly visible light transparent composite films for potential industrial applications.

## 2. Experimental section

### 2.1. Materials and chemicals

The following were used: vanadyl acetylacetonate (C_10_H_14_O_5_V, 98% AR, purchased from Aladdin Reagent Co. Ltd, Shanghai, China), tungsten(VI) chloride (WCl_6_, 98% AR, purchased from Aladdin Reagent Co. Ltd, Shanghai, China), methanol (CH_4_O, 99.5% AR, purchased from Chinese Sinopharm Chemical Reagent Co. Ltd, Shanghai, China), trimethoxymethylsilane (C_4_H_12_O_3_Si, 98% AR, purchased from Aladdin Reagent Co. Ltd, Shanghai, China), oxalic acid dihydrate (C_2_H_4_·2H_2_O, 99.5% AR, purchased from Aladdin Reagent Co. Ltd, Shanghai, China), ammonia solution (NH_3_, 25%~28% AR, purchased from Chinese Sinopharm Chemical Reagent Co. Ltd, Shanghai, China).

### 2.2. Preparation of tungsten-doped VO_2_ films

The VO_2_ precursor was prepared using a sol–gel method, followed by a typical synthesis procedure. Then, 1.00 g commercial vanadyl acetylacetonate and quantitative WCl_6_ were dissolved in 30 mL methanol with vigorous constant stirring in a 50 mL glass beaker for 24 h to ensure that VO(acac)_2_ was dissolved and formed a dark brown solution with a concentration of 0.125 mol·L^−1^. The VO_2_ precursor was formed after aging for 48 h. Simultaneously, fused quartz with the dimensions of 2 cm × 2 cm × 1 mm was prepared after cleaning consecutively with deionized (DI) water, ethanol, and acetone. This fused quartz was used as the substrate for depositing VO_2_ films. The VO_2_ precursor was spin-coated on the fused quartz with a low rate of 800 rpm for 9 s and a high rate of 2500 rpm for 30 s. Then, the sample was dried in a vacuum oven at 100 °C for 30 min to remove excess solvent. The deposition processes of spin-coating and drying in a cyclic fashion were repeated three times, which was marked as (VO_2_) *3. Finally, the samples were annealed at 600 °C for an hour under argon atmosphere through a programmed sintering process.

### 2.3. Preparation of SiO_2_ gels

Gels were obtained via a two-step acid-base catalysis mechanism. In this process, 1 mL of trimethoxymethylsilane and 0.5 mL of 0.001 M oxalic acid solution were dissolved in 10 mL methanol with vigorous constant stirring for 3 h. After hydrolysis, 0.61 mL of 11.2 M ammonium hydroxide solution was added dropwise to the reaction mixture to catalyze the condensation reaction. Then, the reaction mixture was stirred for 30 min, and the solution was left for gelation and aging for 24 h at room temperature.

### 2.4. Preparation of SiO_2_/VO_2_ composite films

The resulting gels were diluted and homogenized with 25 mL methanol using an ultrasonic liquid homogenizer for 30 min. The obtained homogenized solutions were spin-coated on fused quartz for different times (n) in which VO_2_ films had been successfully spin-coated at a low rate of 1000 rpm for 9 s and a high rate of 3000 rpm for 30 s, then the SiO_2_/VO_2_ composite films were marked as (SiO_2_) *n /(VO_2_) *3. Then, the samples were dried in a vacuum oven at 100 °C for 30 min to remove excess solvent and organic groups. The deposition processes of spin-coating and drying in a cyclic fashion were repeated three times. Finally, the samples were annealed at 450 °C for an hour under argon atmosphere through a programmed sintering process.

### 2.5. Characterization

The surface morphologies of the composite films were studied using a JSM-5610LV scanning electron microscope (SEM) (JEOL, Akishima-shi, Tokyo, Japan) at an accelerating voltage of 20 kV. All the samples were coated with gold using a commercial sputtering apparatus prior to SEM measurements. The ultraviolet (UV)–visible diffuse reflectance spectra of the SiO_2_/VO_2_ composite films were measured with a UV–visible spectrophotometer (UV-3600, Shimadzu Corporation, Tokyo, Japan) at normal incidence. The samples obtained were characterized via X-ray diffraction (XRD) using a D/MAX-IIIC X-ray diffractometer (Akishima-shi, Tokyo, Japan) with Cu Kα radiation (λ = 0.15406 nm) to determine the phase structures and crystallite sizes of the samples obtained. The water contact angle of SiO_2_/VO_2_ composite films was measured via the sessile drop technique using a KRUSS DSA100 (Shanghai Xuan Yi Chong Industrial Equipment Co. Ltd., Shanghai, China) contact angle system, with DI water drops of approximately 15 μL applied to three different spots for each coating. Atomic force microscopy (AFM) images were collected using a Park Systems XE-100 (Suwon, Korea) operating in noncontact mode.

## 3. Results and discussion

### 3.1. Morphologies of tungsten-doped VO_2_ films and SiO_2_/VO_2_ composite films

**Figure 1.**
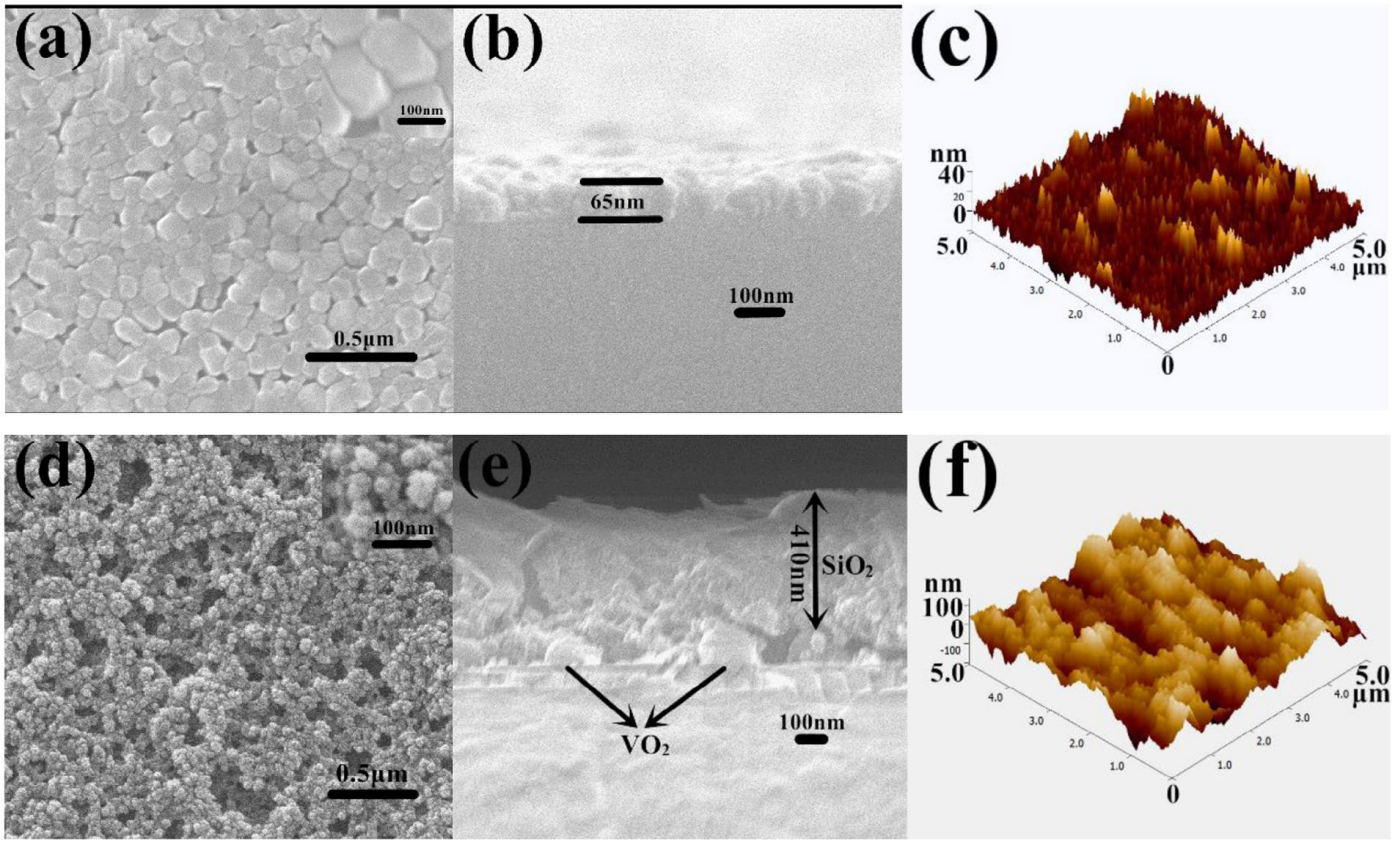
SEM and AFM images of tungsten-doped VO_2_ films (a)–(c) and SiO_2_/VO_2_ composite films (d)–(f) on quartz after calcination.

Figure 1 shows the SEM and AFM images of the surface morphologies of tungsten-doped VO_2_ films and SiO_2_/VO_2_ composite films on quartz after calcination. For tungsten-doped VO_2_ films, the mean grain size and thickness of tungsten-doped VO_2_ films deposited on the quartz substrate were approximately 100 nm (Figure 1(a)) and 65 nm (Figure 1(b)), respectively. The surface roughness value of tungsten-doped VO_2_ was 20.5 nm (Figure 1(c)). Most of the particles were spherical, whereas others were larger oval. The particles were dispersed evenly and densely on the surface. The smallest particle was approximately 50 nm, whereas the largest was approximately 150 nm. We calculated grain size according to the Debye–Scherrer formula [44,45]. The particle sizes of tungsten-doped VO_2_ nanoparticles with different volumes of tungsten, calculated from the width of the 110 reflection, ranged from 20 to 120 nm [26]. We chose a suitable thickness, namely, three layers of spin coating, to improve the optical effect. Increasing the thickness of tungsten-doped VO_2_ films can strengthen the IR-regulating capability of composite films. However, the transmittance in the visible region is significantly reduced [46]. Nevertheless, parts of the surface were still visible, which indicated that the substrate was not fully covered. The optical effect will be enhanced if particle surface coverage is improved. As shown in Figure 1(d), the surface morphologies of SiO_2_/VO_2_ composite films have highly porous networks. We can obtain porosity by controlling the amount of methanol. The annealed tungsten-doped VO_2_ spherical particles and SiO_2_ film were marked in the electron micrograph. The size of the spherical VO_2_ particles is approximately 100 nm, which is consistent with the results of the SEM test and the theoretical calculations. The thickness of SiO_2_ film is approximately 410 nm, and 478 nm is the total film thickness (Figure 1(e)). The surface roughness value of SiO_2_/VO_2_ composite films is 44.0 nm (Figure 1(f)), which is higher than that of tungsten-doped VO_2_ films. The SiO_2_ nanoparticles are relatively uniform, and particle size is smaller than that of VO_2_. The results obtained suggest that the voids between spherical tungsten-doped VO_2_ particles have been filled with SiO_2_ nanoparticles.

### 3.2. IR-regulating property of VO_2_ films and SiO_2_/VO_2_ composite films

**Figure 2.**
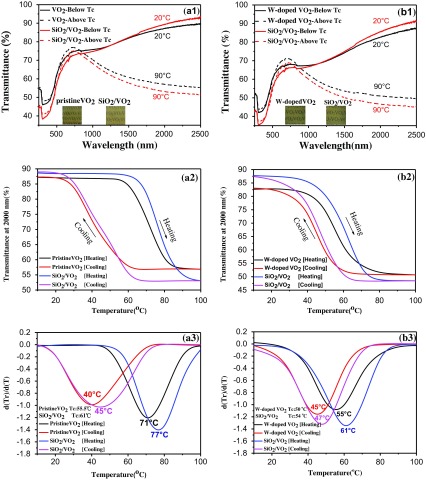
Transmittance spectra (a1), hysteresis loops at 2000 nm (a2), (a)--(c)corresponding d(Tr)/d(T)&T curve (a3) for pristine VO_2_ and SiO_2_/VO_2_ composite films. Transmittance spectra (b1), hysteresis loops at 2000 nm (b2), and corresponding d(Tr)/d(T)&T curve (b3) for tungsten-doped VO_2_ and SiO_2_/VO_2_ composite films.

**Figure 3.**
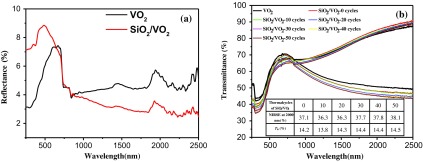
Reflectance spectra for pristine VO_2_ and SiO_2_/VO_2_ composite films (a). Transmittance spectra for SiO_2_/VO_2_ composite films subjected to thermal cycling (b).

Figure 2(a1) shows the transmittance spectra, hysteresis loops at 2000 nm (a2), and corresponding d(Tr)/d(T)&T curve (a3) for pristine VO_2_ and SiO_2_/VO_2_ composite films, as well as the transmittance spectra (b1), hysteresis loops at 2000 nm (b2), and corresponding d(Tr)/d(T)&T curve (b3) for tungsten-doped VO_2_ and SiO_2_/VO_2_ composite films. As shown in Figure 2(a1) and (b1), all samples change their transmittance spectra upon heating from 20 °C to 90 °C. The metal–insulator transition (MIT) of VO_2_ was observed and accompanied by an abrupt change in IR transmittance. The near-infrared switching efficiency (NIRSE) of SiO_2_/VO_2_ composite films (35.5%) was remarkably higher than that of pristine VO_2_ films (30%), although the SiO_2_/VO_2_ film was thicker. This enhancement can be attributed to two factors. First, the size of SiO_2_ nanoparticles was approximately 20 nm, which was considerably smaller than the size of the visible light wavelength. The degree of decrease in transmittance and loss of light posed negligible effects. The size of SiO_2_ particles was significantly smaller than that of VO_2_ particles, and the voids between spherical VO_2_ particles were filled with SiO_2_ nanoparticles. Nanosilica with small particles provided a coarse micro-nanostructure by stacking the surfaces, thereby promoting reduction in the reflectivity of composite films and improving the total amount of sunlight passing through the composite films, as shown in Figure 3(a). Consequently, the NIR control capability of composite films was enhanced. This result shows potential value in promoting SiO_2_/VO_2_ composite films. To fully display the superior optical properties of SiO_2_/VO_2_ composite films, the visible transmittance (*T*_lum_, 380–780 nm) and solar spectral transmittance (*T*_sol_, 300–2500 nm) of all the films were calculated according to standard spectra [47] and Equation (1) [48] for the prepared VO_2_ thin films and SiO_2_/VO_2_ composite films:(1)
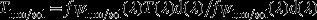



where *T*(λ) corresponds to the transmittance at the transmission wavelength λ, *ψ*_lum_ denotes the standard luminous efficiency function for the photopic vision of the human eyes, and the wavelength range of *T*(λ) measures 380–780 nm; *ψ*_sol_ refers to solar spectral irradiance that corresponds to an atmospheric mass of 1.5. The angle between the sun and the horizon is above 37°, and the wavelength of *T*(λ) ranges from 300 nm to 2500 nm. Luminous transmittance modulation (∆*T*_lum_) and solar energy modulation (∆*T*_sol_) are defined as follows:(2)
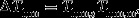

(3)
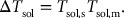



*T*_lum,s_ and *T*_lum,m_ indicate the luminous transmittance of films in semiconductor and metal states, respectively. *T*_sol,s_ and *T*_sol,m_ indicate the solar transmittance of films in semiconductor and metallic states, respectively. MIT temperature was defined as *T*_C_ = (*T*_1_ + *T*_2_)/2, and the width of the hysteresis loop was described by ∆*T* = *T*_1_−*T*_2_. Table 1 presents all the results obtained. ∆*T*_sol_ was 4.6% for pristine VO_2_ film, the hysteresis width (∆*T*_C_) reached 31 °C, and the transition temperature (*T*_C_) was measured to be 55.5 °C, which was lower than the classical 67 °C for pristine VO_2_. These results were attributed to the size effect of nanoparticles: more grain boundaries and film defects occurred with small particle size, and surface energy was sufficiently high to decrease the activation energy and the energy barrier. Thus, the phase change and reverse-phase transition proceeded easily [49,50]. The actual phase transition temperature decreased. ∆*T*_sol_ reached 5.1%, the hysteresis width (∆*T*_C_) measured 32 °C, and the transition temperature (*T*_C_) was 61 °C for SiO_2_/VO_2_ composite films. For tungsten-doped VO_2_ films, ∆*T*_sol_ reached 4.8% and 5.9% for VO_2_ films and SiO_2_/VO_2_ composite films, the hysteresis width (∆*T*_C_) measured 10 °C and 14 °C, and the transition temperature (*T*_C_) was 50 °C and 54 °C, respectively, which were considerably lower than the classical 67 °C for pristine VO_2_. Three theories have been proposed to explain the reduced phase transition temperature caused by element doping: theory of length [51], theory of charge transfer [52], and stress theory [53].

**Table 1.** Solar energy modulation parameters of pristine VO_2_ film and SiO_2_/VO_2_ composite films.

W/(W+V) molar ratio (%)		*T*_lum,s_ (%)	*T*_lum,m_ (%)	*T*_sol,s_ (%)	*T*_sol,m_ (%)	∆*T*_sol_ (%)	NIRSE at 2000 nm (%)	*T*_ir_ (%)	∆*T*_C_ (°)	*T*_C_ (°)
0	pristine VO_2_	67.5	68.9	70.2	65.6	4.6	30	12.2	31.1	55.3
	SiO_2_/VO_2_	63.3	65.2	67.3	62.2	5.1	35.5	13.9	32.2	60.6
0.5	pristine VO_2_	64	64.8	64.7	59.5	4.8	31.7	12.2	10.4	50.4
	SiO_2_/VO_2_	60.1	61.2	62.5	56.6	5.9	39	14.9	13.4	54.3

It was obvious that the VO_2_ thin films doped with a metal ion did not change the characteristic of enhancing the near IR control capability of the composite film shown in Table 1. The phase transition temperature of VO_2_ films could be reduced by controlling the amount and type of metal ion. That made it possible to reduce the phase transition temperature and improve the infrared modulation ability for the composite films simultaneously. The aforementioned results demonstrated that SiO_2_/VO_2_ composite films showed excellent thermochromic properties similar to those of VO_2_ films, and the solar modulation capability of VO_2_ films improved significantly. Doping with tungsten significantly affected the phase transition temperature of VO_2_. For example, when doping amount reached 0.5%, the phase transition temperature of the products was decreased to 50 °C. However, when doping amount increased to 1.5% [26], the phase transition temperature was further reduced to 32 °C. Simultaneously, the hysteresis width of the film was reduced effectively by tungsten doping and SiO_2_/VO_2_ composite films.

A thermal cycling test was performed to study the cycling stability of SiO_2_/VO_2_ composite films in terms of IR regulation. The quartz glass coated with SiO_2_/VO_2_ composite films was thermally cycled between 0 °C and 100 °C on a heating platform. The UV transmittance of composite films was measured after every 10 cycles, and the results are shown in Figure 3(b). The NIRSE of SiO_2_/VO_2_ composite films at 2000 nm decreased slightly at 10th and 20th thermal cycles, but showed a marked increase at 30th to 50th cycles. The NIR control capability of composite films also enhanced slightly; however, the overall transmittance of composite films reduced slightly. The UV transmittance of composite films was measured after 24 h, and the NIR transmittance of composite films increased, with a NIRSE of 40.7% (at 2000 nm); meanwhile the NIR control capability was 16%. The enhancement can be attributed to the improved crystallinity of VO_2_ under the condition of continuous thermal cycling, which resulted in a better infrared modulation.

### 3.3. Anti-oxidation and anti-acid properties of SiO_2_/VO_2_ composite films

The structures of SiO_2_/VO_2_ composite films effectively enhanced the anti-oxidation and acid-resisting properties of pristine VO_2_ films. SiO_2_ film was used not only used as anti-reflective layer but also as a barrier for oxygen diffusion to effectively prevent VO_2_ from being oxidized to V_2_O_5_ or other high valence vanadium oxides. This assumption was confirmed by a comparative study of uncoated and coated SiO_2_ nanoparticles after exposing them to air for 1 month at room temperature.

**Figure 4.**
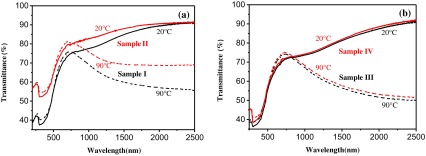
Transmittance spectra of pristine VO_2_ films (a) and SiO_2_/VO_2_ composite films (b) before and after treatment with air for 1 month at room temperature.

As shown in Figure 4(a) and (b), the MIT of pristine VO_2_ films was observed and accompanied by an abrupt change in IR transmittance. The NIRSE of pristine VO_2_ films and SiO_2_/VO_2_ composite films was 34.0% and 35.7% at 2000 nm for Sample I and Sample III, while the NIRSE was 17.8% and 35.5% for Sample II and Sample IV after air exposure for 1 month at room temperature. NIRSE was reduced by about 47.6% for the uncoated VO_2_ films, whereas it changed slightly in SiO_2_/VO_2_ composite films. Evidently, VO_2_ films underwent vanadium oxide reaction and were oxidized to V_2_O_5_ or other high valence vanadium oxides in air [12]. VO_2_ particles can be effectively prevented from being oxidized to other high valence vanadium oxides by adding a layer of SiO_2_ on the VO_2_ film as a barrier layer for oxygen diffusion.

**Figure 5.**
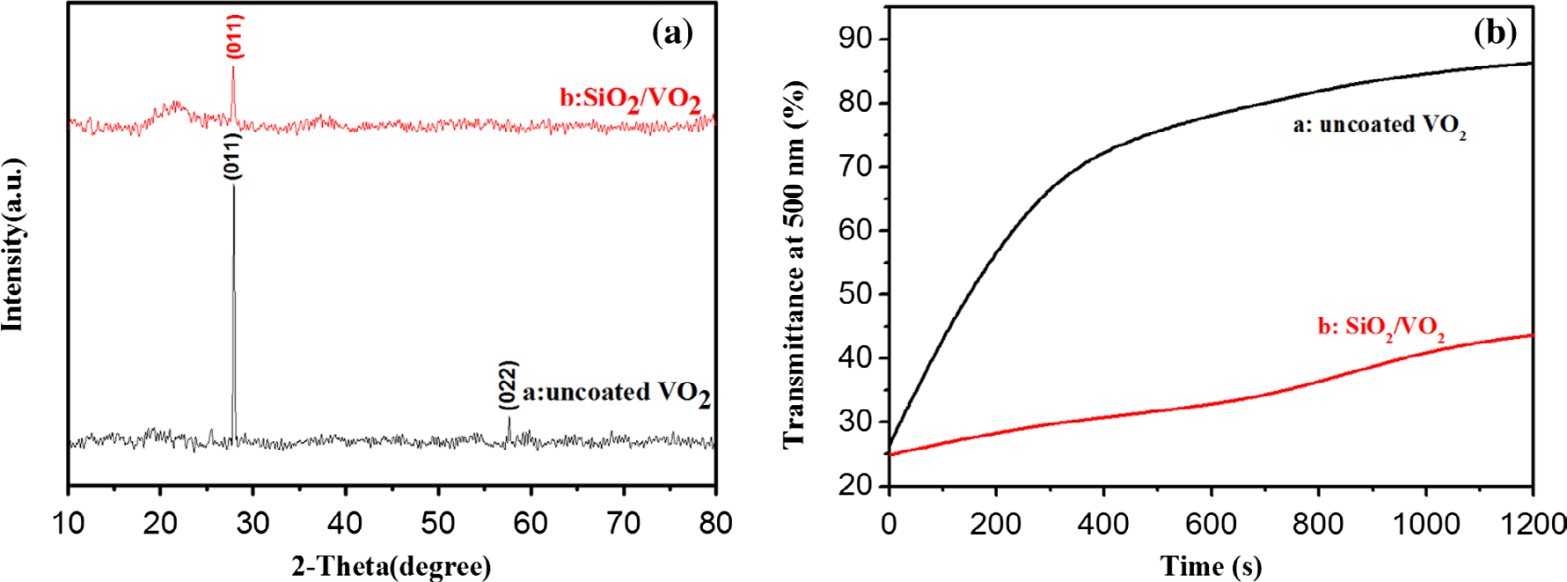
XRD patterns of the pristine uncoated VO_2_ and SiO_2_/VO_2_ composite films (a). Transmittance spectra at 500 nm of the pristine uncoated VO_2_ and SiO_2_/VO_2_ composite films in acidic solution at different times (b).

Figure 5(a) shows the XRD patterns of uncoated VO_2_ films (black line) and SiO_2_/VO_2_ composite films (red line), and Figure 5(b) shows transmittance spectra at 500 nm of uncoated VO_2_ and SiO_2_/VO_2_ composite films in acidic solution at different durations. The XRD signals are relatively weak due to the nanocrystalline structure and small thickness of the films. The peaks at 27.9° and 57.7° were ascribed to the (011) and (022) planes, respectively, of monoclinic VO_2_ (JCPDS No. 82–0661), which were observed in pristine VO_2_ films. However, the peak intensity at 27.9° visibly decreased, and the peak at 57.7° disappeared due to the destruction of the SiO_2_ layer in SiO_2_/VO_2_ composite films. Moreover, no significant diffraction peaks were observed for other vanadium oxides.

An acid corrosion resistance test was carried out to study the corrosion resistance of SiO_2_/VO_2_ composite films. An equal amount of hydrochloric acid solution was added to uncoated VO_2_ and SiO_2_/VO_2_ composite films at room temperature. The hydrochloric acid solution (pH = 1) was selected to accelerate film erosion and to ease observation. During acid erosion, we directly observed that the surface of uncoated VO_2_ films quickly dissolved, and film color changed from dark brown to light yellow. In contrast, SiO_2_/VO_2_ films did not show visible color changes after the first 10 min. The surface of SiO_2_/VO_2_ composite films appeared as light brown patches at 10 to 20 min, thereby indicating that the superhydrophobic SiO_2_ film was partially destroyed leading to the corrosion of VO_2_ film. After 30 min, VO_2_ film was completely dissolved, whereas some unbroken SiO_2_ film remained on the glass substrate surface. Figure 5(b) shows film transmittance at 500 nm after different exposure times to the same acid. The curve clearly shows that the transmittance of uncoated VO_2_ film rapidly increased from 25.7% to 73.0% after 400 s of acidic treatment. However, the transmittance of SiO_2_/VO_2_ composite films increased from 24.7% to 30.8%. The results indicate that SiO_2_/VO_2_ composite films can withstand acidic treatment due to the protection of the SiO_2_ hydrophobic layer.

### 3.4. Superhydrophobic surfaces of SiO_2_/VO_2_ composite films

**Figure 6.**
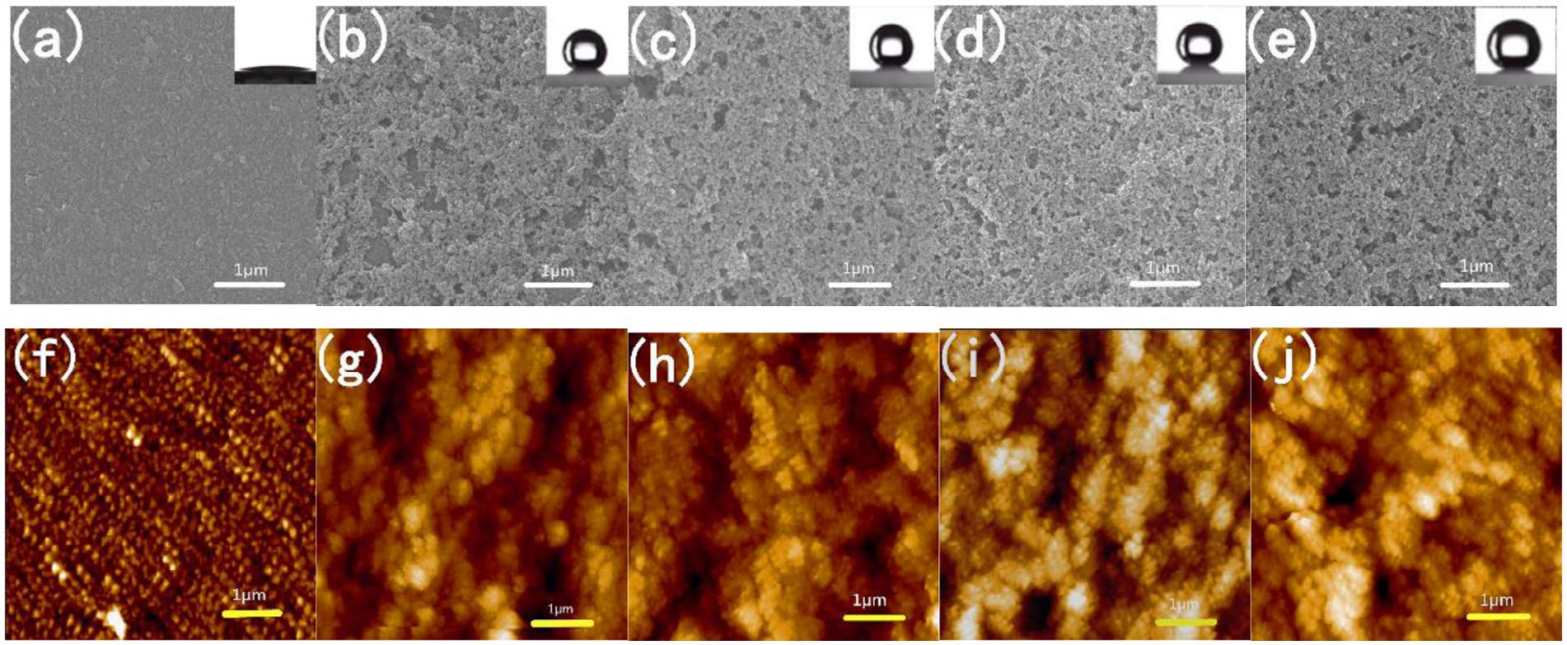
SEM micrographs of VO_2_ (a) and SiO_2_/VO_2_ composite (b)–(e) films. AFM images of VO_2_ (f) and SiO_2_/VO_2_ composite (g)–(j) films on quartz after calcination. (a) (VO_2_) *3, (b) (SiO_2_) *1/(VO_2_) *3, (c) (SiO_2_) *2/(VO_2_) *3, (d) (SiO_2_) *3/(VO_2_) *3, and (e) (SiO_2_) *4/(VO_2_) *3. Insets show images of the water contact angle of the corresponding films.

**Figure 7.**
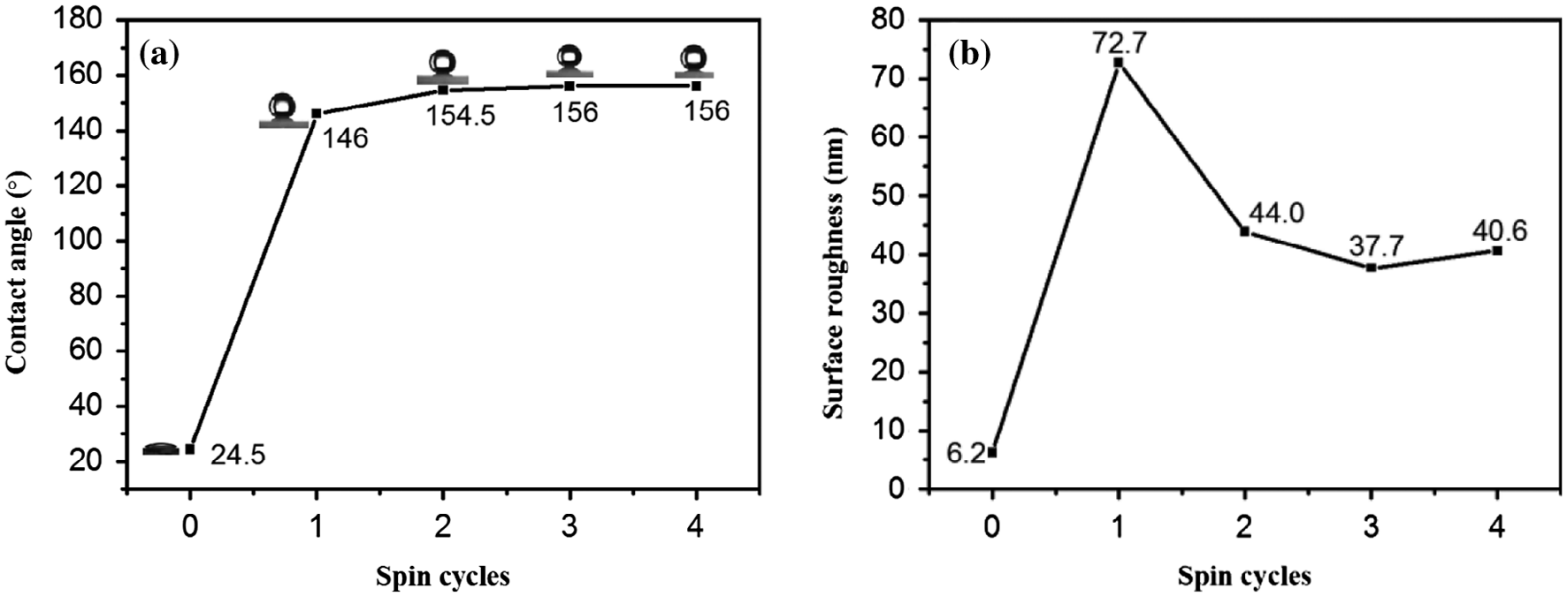
Water contact angles and average roughness of the composite films. The composite films were prepared from (VO_2_) *3, (SiO_2_) *1/(VO_2_) *3, (SiO_2_) *2/(VO_2_) *3, (SiO_2_) *3/(VO_2_) *3, and (SiO_2_) *4/(VO_2_) *3.

Figure 6 shows SEM and AFM images of VO_2_ films and SiO_2_/VO_2_ composite films on quartz after calcination. The SEM images showed that all the membranes featured a highly nanoporous network structure and were deposited with spherical SiO_2_ particles with a size of 20 nm. Porosity was determined by controlling the amount of methanol [54,55]. The water contact angle of the surface is related to both surface energy and roughness [56,57]. The combination of micrometer- and nanometer-sized pores induced superhydrophobicity. Within a certain range, higher porosity resulted in increased static contact angle. Increasing roughness allowed the entrapment of more air between the water and the surface. The surface roughness of the nanostructure composite films decreased with an increase in SiO_2_ layers before gradually stabilizing. As shown in Figure 7(a) and (b), the average surface roughness of pure VO_2_ films was 6.2 nm, which was significantly lower than that of SiO_2_/VO_2_ composite films. Consequently, pure VO_2_ thin films exhibited a smaller water contact angle, which was hydrophilic. The average roughness of SiO_2_/VO_2_ composite films was much higher than VO_2_ thin films, which was sufficient to meet the requirements of roughness of superhydrophobicity. It is well known that methytrimethoxysilane is a common silane coupling agent, and the surface of SiO_2_ nanoparticles (formed during the reaction after the methytrimethoxysilane was decomposed) were modified simultaneously. The combination of micrometer- and nanometer-sized pores and low surface energy induced superhydrophobicity.

The average roughness of SiO_2_/VO_2_ composite films with one spun layer was highest, and it was the most prone to failing full coverage of VO_2_ films. Consequently, the water contact angle was less than 150°. With the increase in the number of spin-coated SiO_2_ layers, the water contact angle of composite films reached 155°, whereas average roughness stabilized at 40 nm, which was highly suitable for the use of transparent superhydrophobic composite films for glazing. However, scattering of light by these films increased remarkably, whereas transmittance was reduced significantly when surface roughness was higher than 100 nm [58]. Hence, it was not conducive to the application and promotion for SiO_2_/VO_2_ composite films.

**Figure 8.**
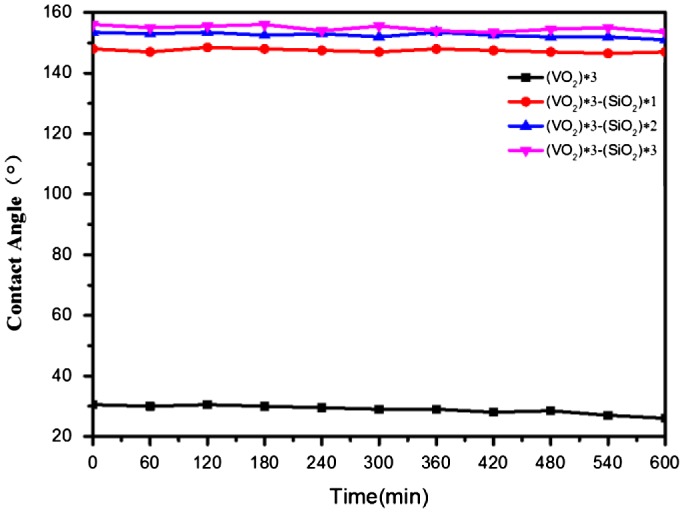
Changes in water contact angle of SiO_2_/VO_2_ composite films during irradiation with 160 mW·cm^−2^ UV light. Composite films were prepared from (VO_2_) *3 (squares), (SiO_2_) *1/(VO_2_) *3 (circular dots), (SiO_2_) *2/(VO_2_) *3 (blue triangles), and (SiO_2_) *3/(VO_2_) *3 (pink triangles).

The UV stability of superhydrophobic surface was observed by comparing changes in the water contact angle on the coating surface. Figure 8 displays changes in the water contact angle of superhydrophobic SiO_2_/VO_2_ composite films under 365 nm illumination with an intensity of 160 mW^**.**^cm^−2^. The water contact angle of VO_2_ films was initially 30.5° and was hydrophilic, whereas the water contact angle of (SiO_2_) *1/(VO_2_) *3 reached 148° for failing to fully cover the VO_2_ film. The water contact angles of (SiO_2_) *2/(VO_2_) *3 and (SiO_2_) *3/(VO_2_) *3 were 152.5° and 155°, respectively. The hydrophobic angles of the four films did not change with prolonging irradiation time with a UV lamp until irradiation time was extended to 10 h. The water contact angle of (VO_2_) *3 film was reduced by 26.5°, and hydrophilicity was enhanced. VO_2_ and TiO_2_ are typical photosensitive semiconductor materials. Films exposed to UV light will produce photogenerated holes. The interaction between photogenerated holes and lattice oxygen will form oxygen vacancies on the surface of VO_2_, and hydrogen bonds will be formed through the reaction of oxygen vacancies and water molecules, thereby enhancing the hydrophilicity of surfaces [59,60]. In contrast, the water contact angles of (SiO_2_) *1/(VO_2_) *3, (SiO_2_) *2/(VO_2_) *3, and (SiO_2_) *3/(VO_2_) *3 changed minimally; the hydrophobic angles were 147°, 152.5°, and 154.5°, respectively. Consequently, SiO_2_/VO_2_ composite films were not only superhydrophobic but also resistant to high-intensity UV illumination.

## 4. Conclusions

In this work, highly transparent, IR-regulating and superhydrophobic nanostructured SiO_2_/VO_2_ composite films were fabricated via a sol–gel approach. The composite films comprised an underlying IR-regulating VO_2_ layer and a top protective layer that consists of SiO_2_ nanoparticles. The composite films not only possessed excellent optical properties but also good hydrophobic features. The transmittance of the visible region was higher than 60%, which sufficiently meets requirements of glass lighting. The NIRSE reached 40% at 2000 nm, and the NIR control capability of the composite films was enhanced relatively by 13.9% and 22.1%, whereas the solar modulation capability was enhanced by 10.9% and 22.9%, respectively, in comparison with VO_2_ films and tungsten-doped VO_2_ thin films. The water contact angle of SiO_2_/VO_2_ was higher than 150°. The transparent superhydrophobic surfaces exhibited a high stability to illumination—all the films retained their initial superhydrophobicity even after exposure to 365 nm light with an intensity of 160 mW^**.**^cm^−2^ for 10 h. This composite structure improved the anti-oxidation and anti-acid properties of VO_2_ films. The performances observed are advantageous for surface anti-fogging, rainproofing, and self-cleaning effects of the films studied. Our technique offers a simple and low-cost process for developing stable and visible light transparent superhydrophobic surfaces for industrial applications.

## Disclosure statement

No potential conflict of interest was reported by the authors.

## Funding

The work was supported by the National Natural Science Foundation of China [51572072; 21402045] and the PhD Programs Foundation of the Ministry of Education of China [20114208110004]. This work was also financially supported by the Wuhan Science and Technology Bureau of Hubei Province of China [2013010501010143], the Educational Commission of Hubei Province of China [D20141006], and the Department of Science and Technology of Hubei Province of China [2015CFA118; 2013CFA005].
